# Polarization‐Dependent 3D Holography Generated by Inverse Design Nanoprinting Metasurface

**DOI:** 10.1002/advs.202519147

**Published:** 2025-12-22

**Authors:** Lingxing Xiong, Jintao Gong, Fei Zhang, Wenhao Miao, Qiong He, Dapeng Zhang, Yangjian Cai, Nan Chi

**Affiliations:** ^1^ Key Laboratory for Information Science of Electromagnetic Waves (MoE) Fudan University Shanghai 200433 China; ^2^ National Key Laboratory of Optical Field Manipulation Science and Technology Chinese Academy of Sciences Chengdu 610209 China; ^3^ College of Materials Sciences and Opto‐Electronic Technology University of Chinese Academy of Sciences Beijing 100049 China; ^4^ Research Center on Vector Optical Fields Institute of Optics and Electronics Chinese Academy of Sciences Chengdu 610209 China; ^5^ Shandong Provincial Engineering and Technical Center of Light Manipulation, School of Physics and Electronics Shandong Normal University Jinan 250014 China

**Keywords:** high efficiency gradient descent algorithm, high throughput nanoprinting metasurface, polarization‐dependent 3D display

## Abstract

3D holographic imaging holds great promise as an immersive display platform, enabling the projection of cubic objects in a specific space and facilitating direct interaction with humans. As 3D holographic imaging continues to evolve, metasurfaces emerge as a promising solution due to their lightweight, compact, and small‐scale properties at the macro level. However, to meet the growing demands for higher information storage, greater freedom in display, and smoother 3D holographic imaging, there is a need for a new metasurface capable of accommodating enhanced 3D holographic abilities by incorporating additional freedom. This paper proposes the use of polarization to add imaging freedom and applies modern gradient descent algorithms to accelerate the design of polarization‐dependent 3D holographic imaging. To enable large‐scale fabrication of the designed metasurfaces and bring polarization‐dependent 3D holographic metasurfaces closer to commercialization, a high‐refractive‐index TiO_2_ particle‐doped resin‐based large‐scale nanoprinting method is introduced. The design's capabilities are demonstrated by achieving 24 3D holographic images at four polarizations in simulation and ten 3D holographic images in experiment at two polarizations. This work paves the way for the commercialization of multi‐dimensional 3D holographic imaging based on metasurfaces, ushering in a new era of compact, lightweight, and large‐scale multi‐dimensional 3D holographic displays.

## Graphical abstract

A novel polarization‐dependent 3D holography is proposed by introducing polarization as an additional freedom, enabling enhanced depth selectivity and greater control over holographic reconstruction. The efforts perfectly combine the polarization and 3D holography display into ADAM gradient descent algorithm, the application of nanoprinting further makes the polarization‐dependent 3D holography metasurface functional.

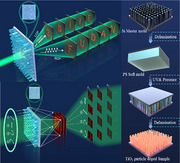

## 1 Introduction

Polarization‐dependent multi‐depth holography is an advanced optical technique that leverages the polarization state of light to encode and reconstruct 3D images across multiple depth planes. By combining the principles of holography and polarization optics, this method introduces an additional degree of freedom, enabling enhanced depth selectivity and greater control over holographic reconstruction. The interplay of light polarization and depth encoding opens new possibilities for achieving high‐resolution and multidimensional imaging.

The core principle of this technique lies in utilizing polarization‐sensitive elements, such as birefringent materials,^[^ ^1^ ^]^ metasurfaces,^[^ ^2^, ^3^, ^4^, ^5^, ^6^, ^7^, ^8^, ^9^ ^]^ or spatial light modulators,^[^ ^10^, ^11^, ^12^ ^]^ to differentiate holographic information encoded at various depths. By tailoring the polarization state—linear, circular, or elliptical—light waves interact with the optical components in a manner that selectively encodes specific depth layers. During reconstruction, these polarization states can be analyzed to retrieve depth‐specific holographic information, offering unprecedented control over the spatial and depth characteristics of the holographic image.

Applications of polarization‐dependent multi‐depth holography span diverse fields, including augmented reality (AR), optical encryption,^[^ ^13^, ^14^, ^15^ ^]^ and biomedical imaging.^[^ ^16^ ^]^ In AR displays, which provide realistic depth cues by dynamically controlling polarization‐based depth planes.^[^ ^17^, ^18^, ^19^, ^20^ ^]^ For optical encryption, the added layer of polarization‐dependent encoding enhances security and complexity.^[^ ^21^, ^22^, ^23^, ^24^, ^25^, ^26^ ^]^ In medical and biological imaging, this technique enables non‐invasive,^[^ ^27^ ^]^ high‐resolution visualization of layered tissue structures,^[^ ^28^ ^]^ making it an invaluable tool for both diagnostics and research. This innovative approach marks a significant step forward in the evolution of holographic technologies, promising transformative impacts in science and technology.

For the wide application of polarization‐dependent multi‐depth holography, metasurface undoubtedly is a promising candidate for undertaking the polarization‐dependent multi‐depth holography,^[^ ^29^, ^30^ ^]^ its lightweight and compact properties instead of heavy and bulky conventional optics make it a powerful manipulator for electromagnetic (EM),^[^ ^31^ ^]^ in the previous studies, metasurface could be served to manipulate a lot of properties of EM in the visible region, such as amplitude,^[^ ^32^, ^33^ ^]^ phase,^[^ ^34^, ^35^, ^36^ ^]^ orbit angular momentum,^[^ ^37^, ^38^, ^39^, ^40^ ^]^ polarization,^[^ ^41^ ^]^ frequency,^[^ ^42^ ^]^ with the variation of the size of each metaatom on metasurface, both transmission phase and geometry phase^[^ ^43^ ^]^ could be introduced and multi EM properties could be manipulated, its remarkable EM regulation ability in the visible region has applications in many fields, such as beam focusing,^[^ ^44^ ^]^ beam deflection,^[^ ^45^ ^]^ far‐field holography,^[^ ^46^, ^47^, ^48^, ^49^, ^50^, ^51^ ^]^ nearfield printing^[^ ^52^ ^]^ and structural color display,^[^ ^53^, ^54^ ^]^ thus metasurface is a proper device which could combine polarization and 3D farfield holography.^[^ ^55^ ^]^ Compared with traditional optical manipulation device applied in visible region, such as liquid crystal, its usually larger pixel size (>1.08 µm) induce the narrow viewing angle less than 8°, limiting the application in 3D holography,^[^ ^56^ ^]^ with the subwavelength designed metasurface which could make the viewing angle easily reach 22° in 3D holography, thus the metasurface is more adequate to achieve 3D holography with its small volume and large manipulation freedom.

For the fabrication, a proper fabrication method should be selected to make wide use of the polarization‐dependent 3D holography based on the metasurface. Compared with the traditional fabrication method of the metasurface, because of the subwavelength structure in designing the metasurface in the visible region, a sub‐100 nm structure could be easily demanded. To obtain the sub 100 nm structure, traditionally electron beam lithography (EBL),^[^ ^57^ ^]^ focusing ion beam lithography^[^ ^58^ ^]^ and other fabrication methods, such as SP lithography,^[^ ^59^ ^]^ but these methods need high voltage and high vacuum, and the patterns only formed point by point, these time‐consuming ways show adverse effects on high throughput fabrication of polarization‐dependent 3D holography metasurface, further leading to the small scale application of polarization‐dependent 3D holography metasurface. With the development of large scale fabrication technology, the nanoprinting (NIL) emerges as a promising fabrication technology which could achieve high throughput of metasurface,^[^ ^60^ ^]^ the high refractive index resin is cured by different ways paves the way for the wide use of metasurface in the visible regime.

**Figure 1:**
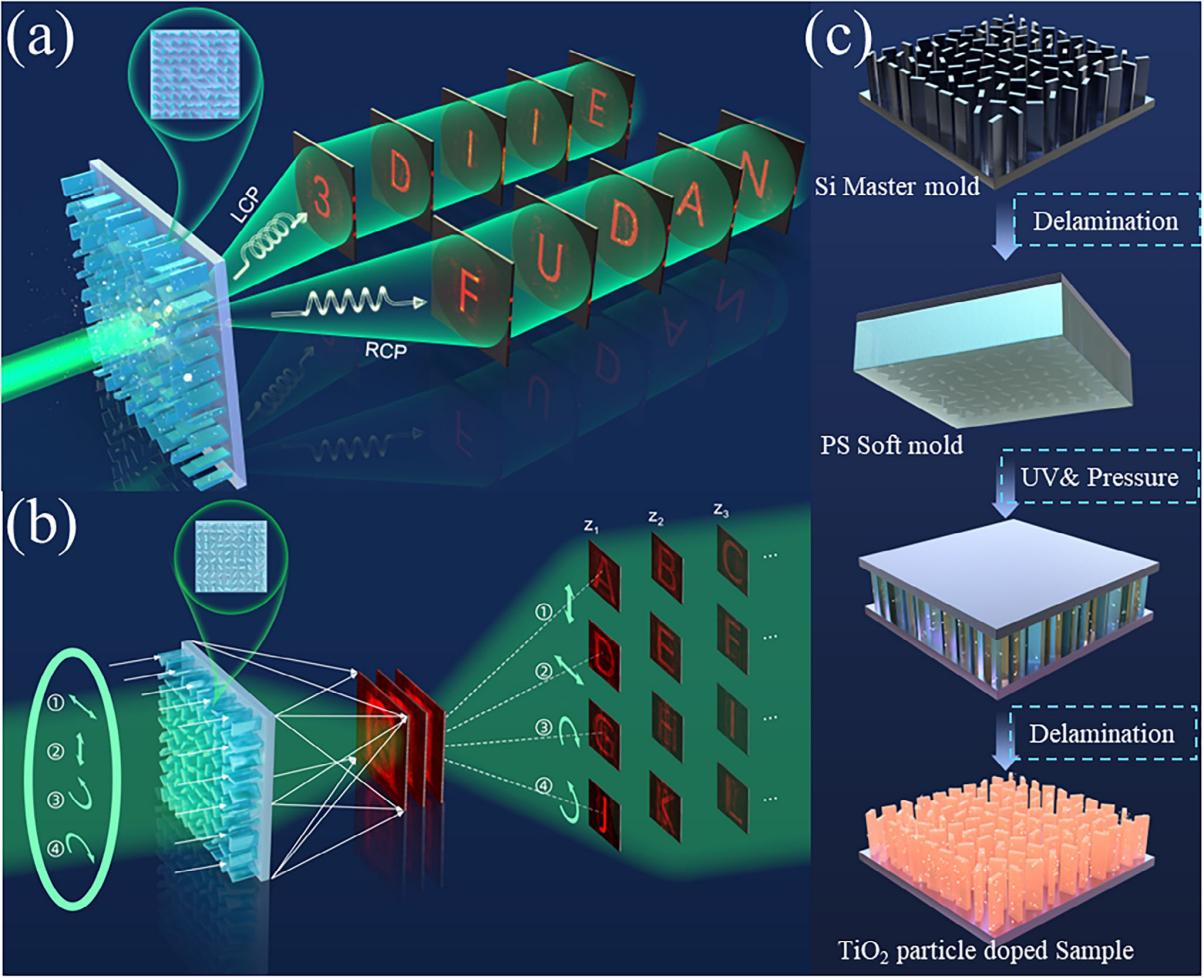
Conceptual illustration of high‐throughput TiO_2_ particle‐doped nanoprinting fabricated polarization‐dependent 3D displays. a) Illustration of 3D holographic imaging based on nanoprinting metasurface with LCP, RCP incident. b) Illustration of 3D holographic imaging based on nanoprinting metasurface with LCP and RCP, 0° linearly polarized (LP), 90° LP incident and RCP, LCP, 90° LP, 0° LP filter. c) The processing flow of TiO_2_ particle‐doped resin‐based nanoprinting metasurface.

In this paper, we designed the polarization‐dependent 3D holography metasurface by gradient descent algorithm, we first design the three holographic images under left circular polarization (LCP) illumination and one holographic image under right circular polarization (RCP) illumination by point source method and verified by nanoprinting metasurface, with the requirement of the increase of information storage capability, gradient descent descent algorithm is introduced to acquire more display channels in 3D space, as shown in **Figure** **1**a. To make the 3D display more vivid at different polarizations, as illustrated in Figure 1a and Figure 1b, more holographic images were designed and experimentally verified. Consequently, we verified the four polarization‐dependent 3D holography with 24 channels in simulation, and more polarization states 3D holographic display could be realized with the help of both the modern gradient descent algorithm and the high‐throughput nanoprinting method. Our efforts perfectly combine the polarization and 3D holography display into the Adaptive Moment Estimation (ADAM) gradient descent algorithm, the application of nanoprinting further makes the polarization‐dependent 3D holography metasurface functional. We are very confident that our efforts could make the commercialization of multifunction display metasurface and promote the development of the display field.

## 2 Results and Discussion

### 2.1 Single‐Step Printing Platform for Polarization‐Dependent 3D Holography Metasurface

3D display based on metasurface technology offers an effective approach to vividly present cubic objects in three‐dimensional space. To enhance the information storage capacity of a single metasurface, extensive efforts have been made to add the additional properities to increase the number of channels. Building upon this foundation, we employed polarization states, LCP and RCP, as an additional degree of freedom to incorporate holographic images into the 3D display. Utilizing the ADAM gradient descent algorithm, we achieved five distinct holographic images at various propagation distances without polarization. Subsequently, by integrating polarization into the design, we successfully obtained the transmission and geometry phase profile for polarization‐dependent 3D holography.

To realize polarization‐dependent 3D holography using metasurfaces, traditional materials commonly employed in the visible regime were explored. Materials such as Si_3_N_4_, GaN, and TiO_2_ are well‐known for their excellent optical manipulation capabilities due to their high refractive index in the visible spectrum. However, the fabrication of metasurfaces with characteristic sizes below 100 nm often involves high‐cost, low‐efficiency processes, including pattern formation and etching. The commercialization of polarization‐dependent holographic metasurfaces requires a material that maintains a relatively high refractive index while being suitable for large‐scale fabrication. A promising solution is the use of high‐refractive‐index particles mixed into a resin, enabling high‐throughput UV‐based nanoprinting. TiO_2_, with a refractive index of 2.6678 at 532 nm, is the most commonly used material for visible metasurfaces. Thus, incorporating TiO_2_ particles into nanoprinting resin presents an effective approach to addressing large‐scale production challenges. Consequently, a TiO_2_ polymer composite, specifically IOC‐133 provided by Inkron (NAGASE Group), was selected as the metasurface material. The fabrication flowchart is illustrated in Figure 1c.

### 2.2 Design Principle of Polarization‐Independent and Polarization‐Dependent 3D Holography

**Figure 2:**
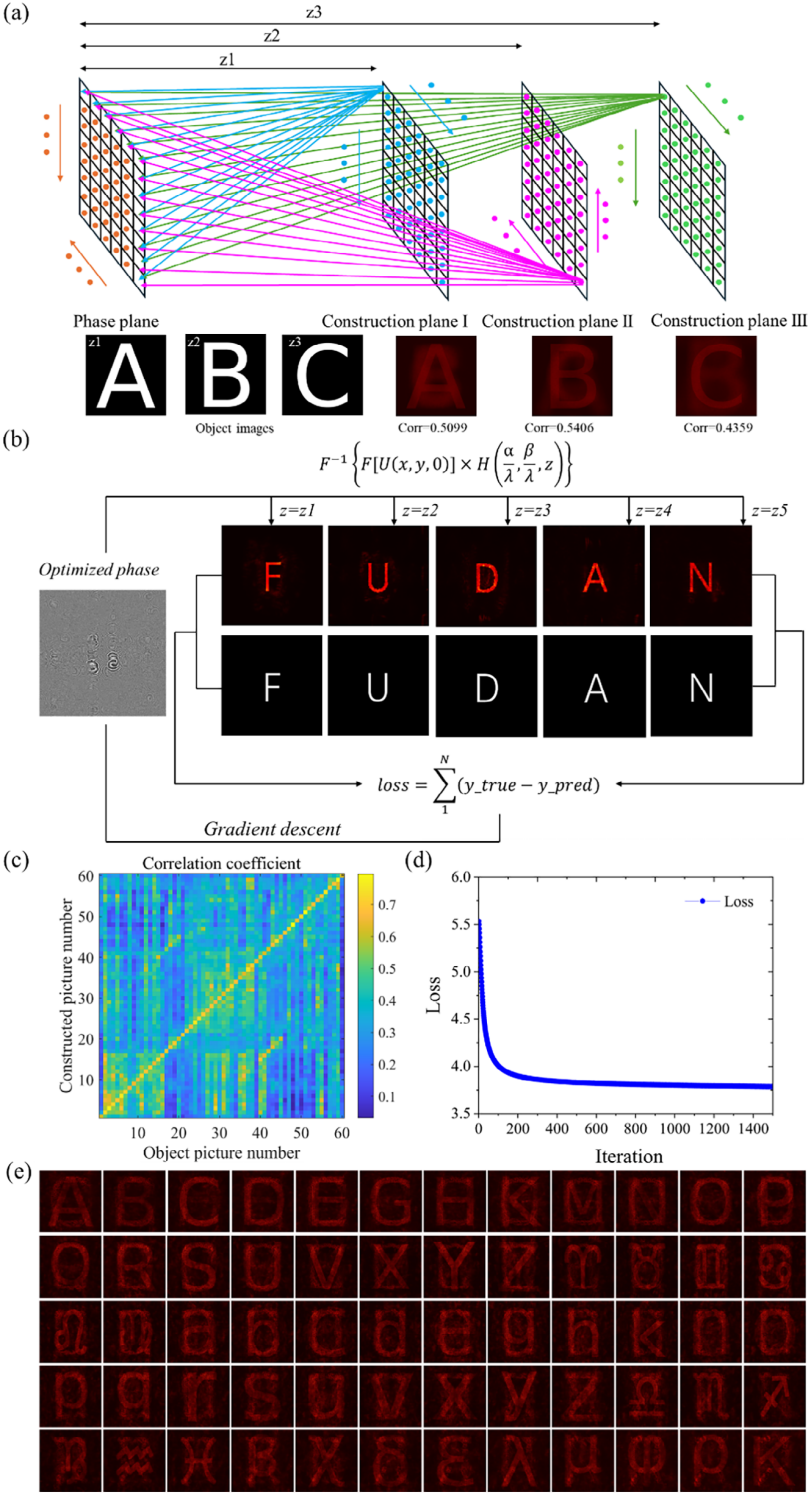
The phase profile optimization flow to realize 3D holography by the point source method and the gradient descent algorithm, respectively. a) The phase profile optimization flow of 3D holography by the point source method, and *z_1_* to *z_3_* are set as 3 , 4 , 5 mm. b) The phase profile optimization flow of 3D holography by the gradient descent algorithm. c) The correlation coefficient between simulated and target images in 60‐channel 3D holography. d) The loss map during the training process for 60‐channel 3D holography by the gradient descent algorithm. e) The simulated 3D holographic images for 60‐channel 3D holography by the gradient descent algorithm.

As shown in **Figure** **2**a, we first designed the three individual holographic images of “3”, “D”, IOE logo with the traditional point source method to preliminarily achieve the holographic images constructed at 3 , 4 , and 5 mm, respectively. Each point in every constructed plane with random phase and certain amplitude propagates to the phase plane and the overlapped electric field is formed after all the points in the constructed plane propagate to every point in the phase plane by the angular spectrum method. In our simulation, the pixel number is set as 2160 × 3840, and the constructed holographic images in simulation via point source method is shown in the first row of **Figure 4**a, the clearly display of the holographic images proves that the point source method is powerful in ordinary 3D holographic calculation process, but the just three

3D holographic images with 2160 × 3840 pixels is so few, and it is hard to adapt to the demand of the smooth 3D holographic display in the modern display technology. To show the 3D display capability clearly and make it convenient to compare the correlation with the following deep learning based results, we employed the correlation coefficient between constructed and target images, which is defined as

(1) $$corr\left(T,R\right)=\frac{cov(T,R)}{\sqrt{D\left(T\right)D\left(R\right)}}$$

where *T* and *R* denotes intensity distribution of two images, cov(*T*, *R*) is their covariance, and D(*T*) and D(*R*) are their variances. We set the pixel number of 3500 × 3500 for three construction images, we calculate the correlation coefficient between three simulated holographic images and their corresponding images by point source method, the correction coefficient is 0.5099, 0.5406, 0.4359 for the first, the second, and the third construction holographic image and the corresponding target images. To find a modern design method to create more holographic images for 3D display with fewer pixels. A modern gradient descent method is introduced to optimize the 3D holography display phase profiles and finally 60 individual holographic images were acquired in different propagation distances with the pixel number of 5000 × 5000. First, we design a 3D holographic display using just five construction planes, only five images, “F”, “U”, “D”, “A”, “N” are set as the object images for the five individual holographic images with just 4000 × 4000 pixels. These holographic slices represent different positions (for example, the propagation distance is set as 3 , 4 , 5 , 6 , 7 mm, respectively) of “F”, “U”, “D”, “A”, “N” images. The angular spectrum method was employed to simulate the propagation process. In each iteration, the constructed holographic images are compared with the object images to calculate the loss, and the gradient descent algorithm is applied to make the loss value smaller in the direction of the fastest gradient descent of the loss function. The angular spectrum diffraction equation is expressed as follows:

(2) $$\begin{array}{cc} & U\left(x,y,z\right)=F^{-1}\left\{A\left(\frac{\alpha }{\lambda },\frac{\beta }{\lambda },z\right)\right\}\\ & \;=F^{-1}\left\{F\left\{U\left(x,y,0\right)\right\}\times H\left(\frac{\alpha }{\lambda },\frac{\beta }{\lambda },z\right)\right\} \end{array}$$

The loss function we used in the gradient descent process could be written as:

(3) $$loss=\frac{1}{N}\sum_{i=1}^{N}{\left({\left|U\left(x_{i},y_{i},z\right)\right|}^{2}-{\left|\hat{U}\left(x_{i},y_{i},z\right)\right|}^{2}\right)}^{2}$$

After 1500 iterations, by comparing the original object images with the corresponding diffracted images, only minor differences were observed, demonstrating that 3D holography can be successfully achieved using the gradient descent algorithm, the simulation flow of the gradient descent method is shown in Figure 2b. To further increase the number of holographic images in 3D display, we fix the pixel number of the matrix as 5000 × 5000 and gradually increase the number of the constructed holographic images to 60, as shown in Figure 2e, all the 3D holographic images are clearly shown at propagation distance from 3  to 32.5 mm with the equal interval of 500 µm. To analysis the quality of the constructive holographic images in 3D holographic display with this gradient descent method, we calculate the correction coefficient of 60 channels 3D constructive holographic images with the corresponding object images, the calculation result is shown in Figure 2c. The loss curve of the optimization process is shown in Figure 2d. Although we increase the number of the constructed holographic images to 60 in 3D holographic display, the correction coefficient for every constructed holographic image is also above 0.6, and the loss value decreased to ≈ 3.75 after 1500 iterations, proving the inducing of the gradient descent method in 3D holographic display is an efficient way to make the 3D holographic display more smooth with smaller pixel number and calculation time. Compared with the traditional point source design method, the ADAM gradient descent algorithm avoids the point‐to‐point calculation by the conventional method, thus making the phase optimization efficient. Furthermore, the higher correlation coefficients of holographic images in simulation prove the ADAM gradient algorithm could maintain the imaging quality with high efficiency.

To achieve different holographic 3D displays under LCP and RCP incident, the freedom degree of the metasurface should be fully exploited, which could change the pillar size and the rotation angle simultaneously. When the phase profiles of the holographic 3D display under LCP and RCP are calculated independently, the propagation phase and the rotation angle could be calculated by the following equations:

(4) $$\varphi =\frac{\Phi_{LCP}+\Phi_{RCP}}{2}$$

(5) $$\theta =\frac{\Phi_{LCP}-\Phi_{RCP}}{4}$$

By the simple calculation and making full use of the freedom of the metasurface, the holographic display channel is doubled. To make full use of the size and rotation of the metaatoms on nanoprinting metasurface, the input polarization could be added thus the holographic 3D display could be more confidential, the details for this discussion is shown in chapter 2.4.

To make the polarization‐dependent holographic 3D display into practice, the aforementioned cost‐effectively UV‐NIL is undoubtedly a clever way that combines a powerful manipulation ability of visible light and easy fabrication which only relies on the mechanical pattern transformation after the completion of the Si master mold. To fully exploit the degrees of freedom offered by the metasurface, both the geometric phase and transmission phase are incorporated into the fabrication process, although the geometry‐only metasurface could be achieved by rotating a unit cell by half of the calculated phase, in the gradient descent algorithm, it will not expand extra design freedom, thus in the experiment verification, we calculate the phase profile for LCP and RCP separately and combined them by the Equations (4) and (5).

**Figure 3:**
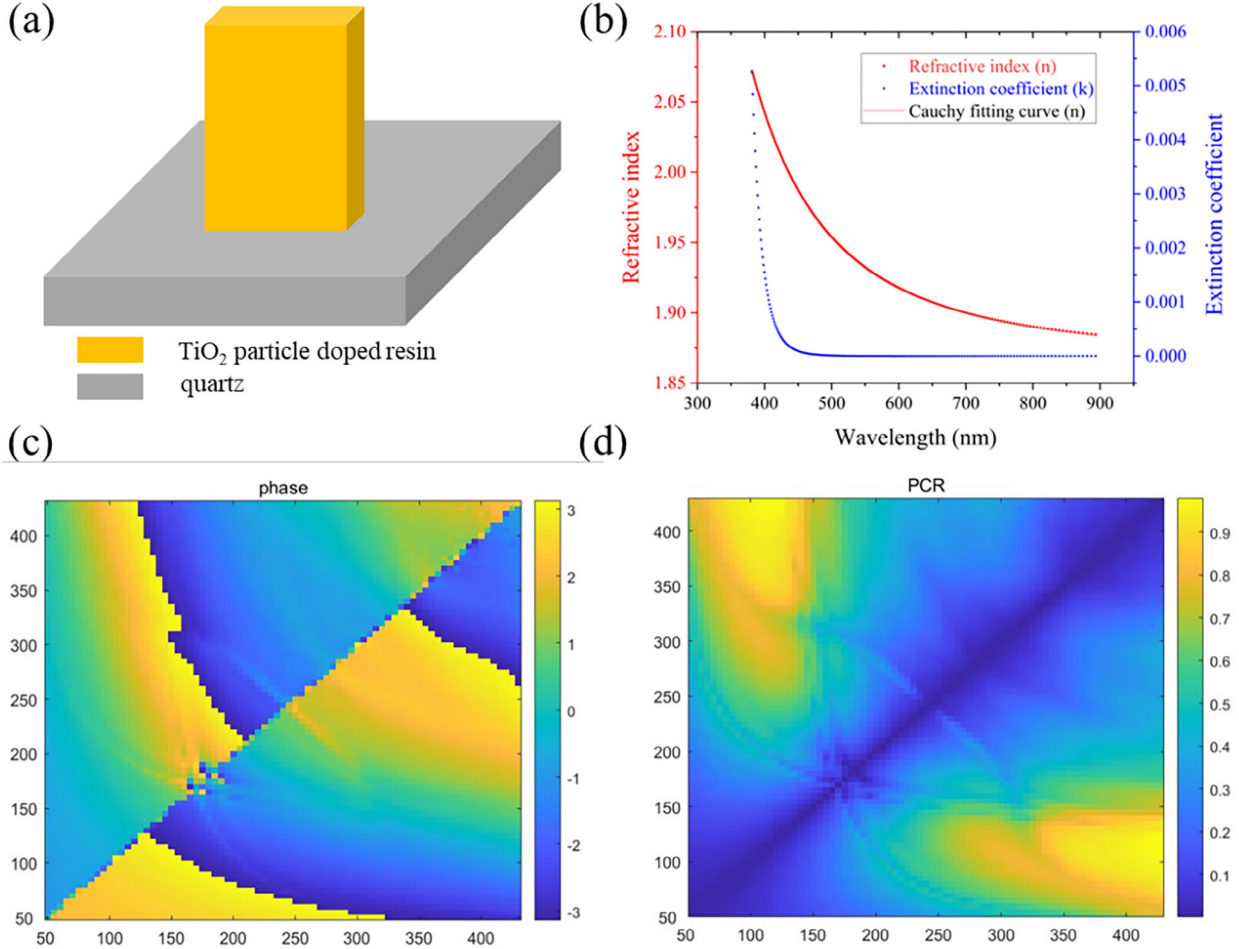
Characteristics of TiO_2_ particle‐doped composited resin‐based nanopillars. a) The structural illustration of the TiO_2_ particle‐doped resin‐based nanoprinted unit pillar, the period is set as 450 nm. b) The refractive index of the TiO_2_ particle‐doped resin, the refractive index at 532 nm is 1.94. c) The transmission phase of the TiO_2_ particle‐based nanoprinted unit cell with the variation of width (W) and length (L) of the nanopillar. d) The polarization conversion ratio (PCR) of the TiO_2_ particle doped resin‐based nanoprinted unit cell with the variation of width (W) and length (L) of the nanopillar.

Thus, eight TiO_2_ nanoparticle‐doped resin nanopillars, capable of covering a full 2π phase modulation range, as well as maintaining a relatively high polarization conversion ratio (PCR), should be selected to construct the polarization‐based 3D metasurface. To obtain these aims, the resin should possess a high refractive index, which not only has powerful optical manipulation ability but easy to be large‐scale fabricated. The TiO_2_ particle‐doped resin is a promising candidate because TiO_2_ has a high refractive index (>2.5) in the visible region. In this work, we use IOC‐133 provided by Inkron (NAGASE Group) as an appropriate metasurface material to achieve polarization‐dependent 3D holography. Composed of an 80% weight ratio of TiO_2_ particles and the polysiloxane matrix, the IOC‐133 provides a high demolding efficiency with its flexibility, water repellency, and low surface tension. To ascertain the refractive index (n) and extinction coefficient (k) of this TiO_2_ particle‐doped resin, a 400 nm thick IOC‐133 thin film is spin‐coated onto a Si substrate. Next, the amplitude ratio (Ψ) and phase difference (Δ) between the s and p components of this thin film are determined through ellipsometry. Finally, the obtained Ψ and Δ data are fitted using the Cauchy dispersion model, which is represented by the following equation:

(6) $$n\left(\lambda \right)=n_{0}+\frac{n_{1}}{\lambda^{2}}+\frac{n_{2}}{\lambda^{4}}$$

where *n* is the refractive index, 𝜆 refers to the wavelength in the units of (nm), and *n*_*0*_, *n_1_*, *n_2_* are the fitting coefficients of the Cauchy model by the measured data, as shown in **Figure** **3**b. From Figure 3b, it can be easily found that Cauchy fitting curve features *n_0_* = 1.867, *n_1_* = 1.059 × 10^4^ (nm^2^), and *n_2_* = 2.800 × 10^9^ (nm^4^) for TiO_2_ nanoparticle‐doped resin used in this work.

To acquire the concrete eight unit cells to undertake the propagation phase and geometry phase manipulation function in the metasurface, the length (L) and width (W) were varied during the optimization sweep, while the period was fixed at 450 nm with the working wavelength of 532 nm. The structure of the TiO_2_ nanoparticle‐doped resin‐based unit cell is illustrated in Figure 3a. Leveraging the high refractive index of the TiO_2_‐composite resin, all unit pillars were optimized to a height of 955 nm using Finite‐Difference Time‐Domain (FDTD) solutions with the fixed length and width of each nanopillar at 400  and 110 nm, respectively.

After performing a width and length sweep of the TiO_2_ nanoparticle‐doped resin‐based nanopillars, the corresponding transmission phase and polarization conversion ratio are presented in Figure 3c,d, respectively. The phase response of eight selected unit cells is presented in Figure S2a (Supporting Information), showing a full 2π phase modulation range with approximately π/4 phase increments between adjacent units. The optimized size of eight unit cells is clearly depicted in Table S1 (Supporting Information), from all these unit cells we acquired eventually, the largest depth‐to‐width ratio is 10.6, thanks to this relatively high aspect ratio and the high refractive index of TiO_2_ nanoparticle doped resin, as shown in Figure 3b, which not only maintain a powerful manipulation ability of electromagnetic fields but facilitate the fabrication of Si master mold and the demolding process in nanoprinting. Although the aforementioned polarization‐based 3D display has only been verified through simulation, the successful fabrication of polarization‐composite unit cells using nanoprinting gives us high confidence that the following ten holographic images from the polarization‐dependent 3D metasurface can be reliably produced through the same method. The high throughput potential of nanoprinting also stimulates the directions to achieve more polarizations‐dependent 3D holography by this nanoprinting metasurface.

### 2.3 Experimental Demonstration of Polarization‐Dependent 3D Holograms by Nanoprinting Metasurface

**Figure 4:**
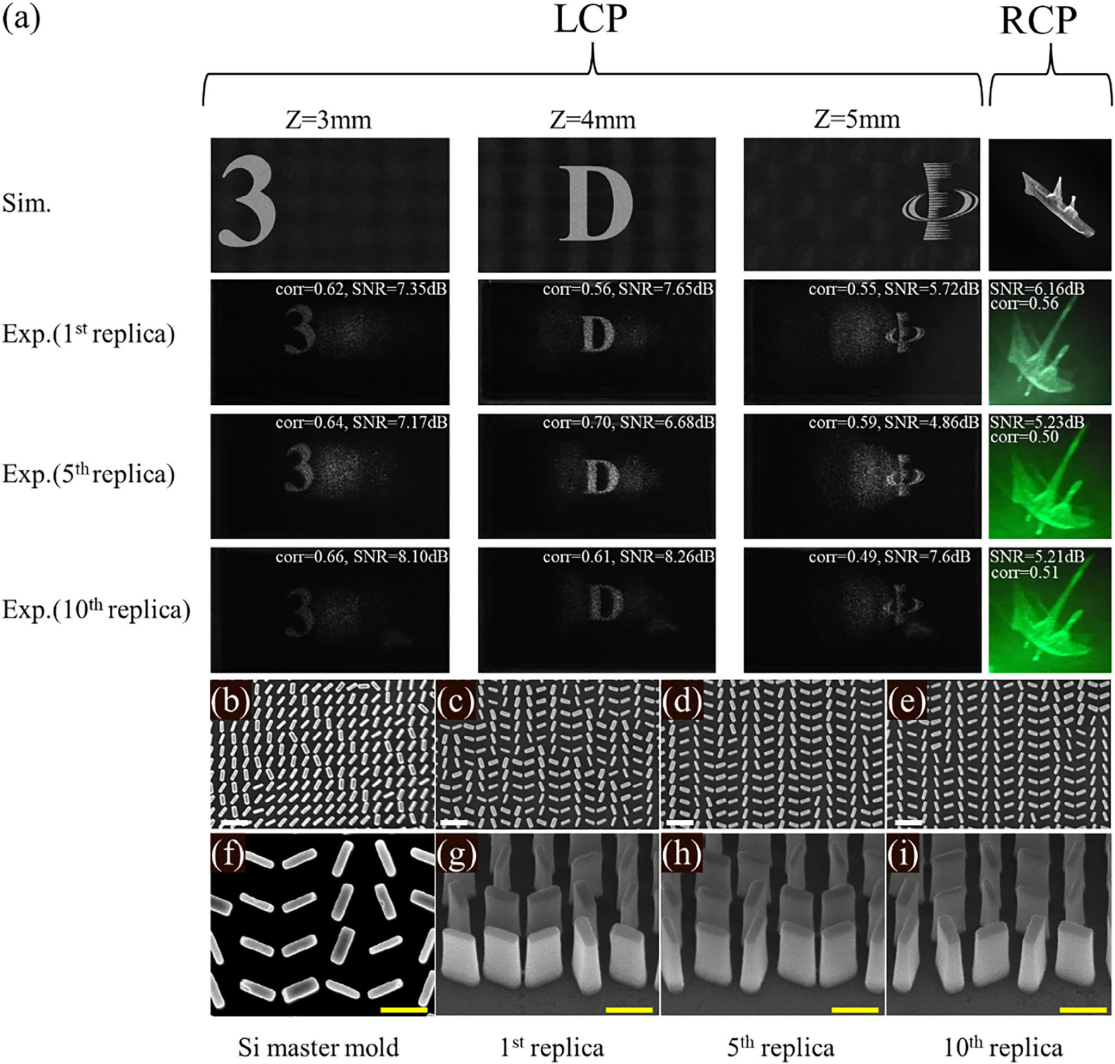
Simulation and experiment results for polarization‐dependent 3D holographic imaging, and scanning electron microscopy (SEM) characterization of the high‐throughput nanoprinting‐based polarization‐dependent metasurface. a) The images in the first row refer to the simulated constructive images at *z*=3 mm, *z*=4 mm, *z*=5 mm under LCP illumination and RCP illumination. The second, third, and fourth rows illustrate the measured constructive images at *z*=3 mm, *z*=4 mm, and *z*=5 mm under LCP illumination and RCP illumination for the 1^st^, 5^th^, and 10^th^ replicas of nanoprinting, respectively. b) Microscopy for the Si master mold at the front view. c–e) The front view and perspective view of the 1^st^, 5^th^, and 10^th^ traditional designed polarization‐dependent 3D nanoprinting metasurface replica, respectively. Note: The white scale bars indicate a length of 1 µm. f–i) Magnified SEM characterization for the Si master mold, the 1^st^, 5^th^, and 10^th^ traditional method designed polarization‐dependent 3D nanoprinting metasurface replicas, respectively. Note: The yellow scale bars indicate a length of 500 nm.

To verify the point source design method and fully show the fabrication ability of high‐throughput nanoprinting, the duration measurements were conducted for polarization‐dependent metasurfaces fabricated by nanoprinting to confirm that all nanoprinted samples exhibited nearly identical performance. As designed, the holographic images of the “3”, “D”, and IOE logos are focused at 3 , 4 , and 5 mm under LCP and RCP illumination respectively from the metasurface, because the designed metasurface has 3840 × 2160 pixels, the size of the holographic images is 1728 µm × 972 µm, thus the object lens is introduced into the optical setups and the three images are accordingly enlarged and captured by charge coupled device (CCD). As depicted in Figure 4a, the first row is the simulation results for polarization‐dependent 3D hologram, for the preliminary verification of polarization‐dependent 3D conception, the holographic image under RCP illumination is designed by the Gerchberg‐Saxton (GS) algorithm, all the constructed images in simulation are clearly distinguished. The measurement results of the first sample are shown in the second row in Figure 4a. Duration tests of the fabrication method were conducted to evaluate the large‐scale, high‐throughput processing capability of the polarization‐dependent 3D holographic metasurface. The 5^th^ and the 10^th^ TiO_2_ nanoparticle doped metasurface transformed from the original Si master mold is measured by the optical setups in Figure S7 (Supporting Information), the experiment results are shown in the third row and the fourth row in Figure 4a, comparing far‐field holographic images of all three nanoprinting samples, almost no difference is found, proving the nanoprinting polarization dependent metasurface could maintain high‐quality imaging in far‐field, benefit from the high‐efficiency nanoprinting method, although the master mold fabricated by EBL is cost consuming, this consuming only happens once. To further assess the holographic imaging quality of the traditional method designed polarization‐dependent 3D holography, the Signal‐to‐Noise Ratio (SNR) is introduced. The SNR of an image is defined as

(7) $$SNR=10\log_{10}\frac{S}{N}\left(dB\right)$$

where *S* represents the average value of the holographic image and *N* denotes the mean background noise. The SNR of the measured holographic images provides a metric to gauge the quality of the polarization‐dependent 3D holography. With the relatively high correlation coefficients (>0.49) and SNR (>4.86 dB) for all experimentally measured polarization‐dependent 3D holography by the traditional design method, the utilization of nanoprinting metasurface to achieve polarization‐dependent 3D holography is initially demonstrated.

To further investigate the pattern transfer capability, which is crucial for the mass production of metasurfaces, we first examine the fabrication of the Si master mold, as shown in Figure 4b. The nanopillar with the designed size, perpendicular to the Si substrate, proving the period and all unit cells we designed have an appropriate size patterned by EBL. After the completion of the Si master mold, the pattern is transformed to the soft substrate PET, forming an inverse morphology composed of polystyrene (PS) on the PET substrate, also known as the soft mold. Finally, the pattern is transformed to the final rigid quartz substrate, where the TiO_2_ particle‐doped resin nanopillars on the quartz substrate are formed and separated from the soft mold through an automated process. Then TiO_2_ particle‐doped resin‐based ten replicas are acquired. Figure 4c–e shows the scanning electron microscope of the 1^st^, 5^th^, and 10^th^ TiO_2_‐doped resin based replicas. To make the comparison the morphology of the Si master mold and the 1^st^, 5^th^, 10^th^ TiO_2_ particle doped resin based replicas, high magnified SEM images for Si master mold, the 1^st^, 5^th^, 10^th^ TiO_2_ particle doped resin based replicas are shown in Figure 4f–i. Although our designed aspect ratio is up to 10.6, the TiO_2_‐doped resin nanopillars maintain clear outlines as designed. Compared with all three replicas, with almost no distortion after the pattern transformed from the soft mold to the TiO_2_‐doped resin layer, the time consumption for fabricating these 10 replicas is less than 15 min. This high fabrication efficiency and perfect polarization‐dependent holographic display undoubtedly pave the way for large‐scale applications of multiplexed 3D display devices.

**Figure 5:**
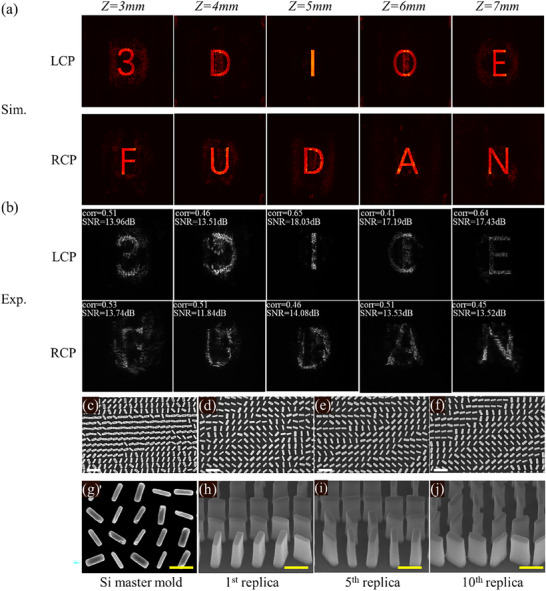
Simulated and experimental results of the ten‐channel polarization‐dependent 3D holography, and SEM characterization of the high‐throughput nanoprinting based polarization‐dependent metasurface. a) The simulated ten holographic images of polarization‐dependent 3D generated by two phase profiles under LCP and RCP illumination. b) The experimentally measured ten polarization‐dependent 3D holographic images generated by nanoprinting metasurface. c–f) The front view SEM images of the Si master mold, the 1^st^, 5^th^, 10^th^ ten‐channel ADAM gradient descent algorithm designed polarization‐dependent 3D holographic nanoprinting metasurfaces. Note: The white scale bars indicate a length of 1 µm. g–j) Magnified SEM characterization of the Si master mold, the 1^st^, 5^th^, and 10^th^ ten‐channel polarization‐dependent 3D holographic nanoprinting metasurface. Note: The yellow scale bars indicate a length of 500 nm.

After the verification of the polarization‐dependent 3D holography based on high‐throughput nanoprinting metasurface with LCP and RCP holography phase profile optimized separately by traditional point source method, with the consideration of increasing the holographic slices within a 3D holography, we conduct the phase optimization by gradient descent algorithm to generate holography images under LCP illumination and RCP illumination separately. In this simulation, the flowchart is the same as Figure 2b, we combined the phase profile to generate 3D holography under LCP and RCP by Equations (4) and (5), the number of holographic images is set as 5 under LCP and RCP illumination, respectively. The capital letter “F”, “U”, “D”, “A”, “N” is used for the object holography images under RCP illumination and the capital letter “3”, “D”, “I”, “O”, “E” is set for the object holography images under LCP illumination. The angular diffraction theory is applied with the propagation distances *z* = 3 , 4 , 5 , 6 , 7 mm, respectively. The loss function is defined as the summation of the difference between the constructed holography images and their corresponding object images. This gradient descent design strategy, undoubtedly different from the traditional individual phase optimization strategy, greatly increases the calculation efficiency at a smaller matrix size. After the 1500 iterations in phase optimization for the polarization‐dependent 3D holography, the constructed holographic images are acquired, as shown in **Figure** **5**a. To verify our gradient descent algorithm‐aided design strategy for polarization‐dependent 3D holography, we fabricate the polarization‐dependent 3D holography nanoprinting metasurface and all ten holographic images are captured by CCD, as shown in Figure 5b, all the measured holographic images are clearly distinguished as designed. As the experimentally measured letter “D” and “O” show a minor blur, it mainly induced by the intrinsic properties of 3D holographic, the light of the adjacent letter “I” between letters “D” and “O” could last for a distance, thus some additional light appears on the construction plane of letters “D” and “O”. To further estimate the holographic imaging quality of all the measured holographic images, we calculated the SNR and correlation coefficient of all measured holographic images. The high correlation coefficient (>0.4) and large SNR (>11 dB) prove that our ADAM gradient descent algorithm designed polarization‐dependent 3D holographic images show great quality in the experiment.

With the verification of large‐scale fabrication, we first complete the Si master mold, after two transformations, the pattern is transformed to the TiO_2_ particle doped resin with the same morphology. The SEM image of the Si master mold with the same morphology as the TiO_2_ particle doped resin composited metasurfaces is shown in Figure 5c, the nanoprinted polarization‐dependent 3D holographic metasurface fabricated after 10 times pattern transformations also shows uniform morphology, proving the naoprinting is an efficient way for the commercialization of the polarization‐dependent 10 channels 3D holographic metasurface, as shown in Figure 5d–f. To further display the perfection fabrication of the nanoprinted polarization‐dependent 3D holographic metasurface, the magnified SEM images for the Si master mold and the polarization‐dependent 3D holographic nanoprinted metasurafce after one time, 5 times, 10 times pattern transform from softmold are shown in Figure 5g–j, the clear outlines of all polarization‐dependent 3D holographic metasurface, the almost identical morphology and the totally time consumption less than 15 min for fabricating all these ten nanoprinting metasurfaces prove that the nanoprinting is a high‐efficiency fabrication method to make the polarization‐dependent 3D holography come into practice. Our design and fabrication not only provide a high efficiency optimization method for multiplexing display scenes, but also make the designed nanoprinting metasurface with high throughput, which promotes the metasurface with powerful EM modulation capability commercialized in daily life.

### 2.4 Exploration of Four Polarizations‐Dependent 3D Display

**Figure 6:**
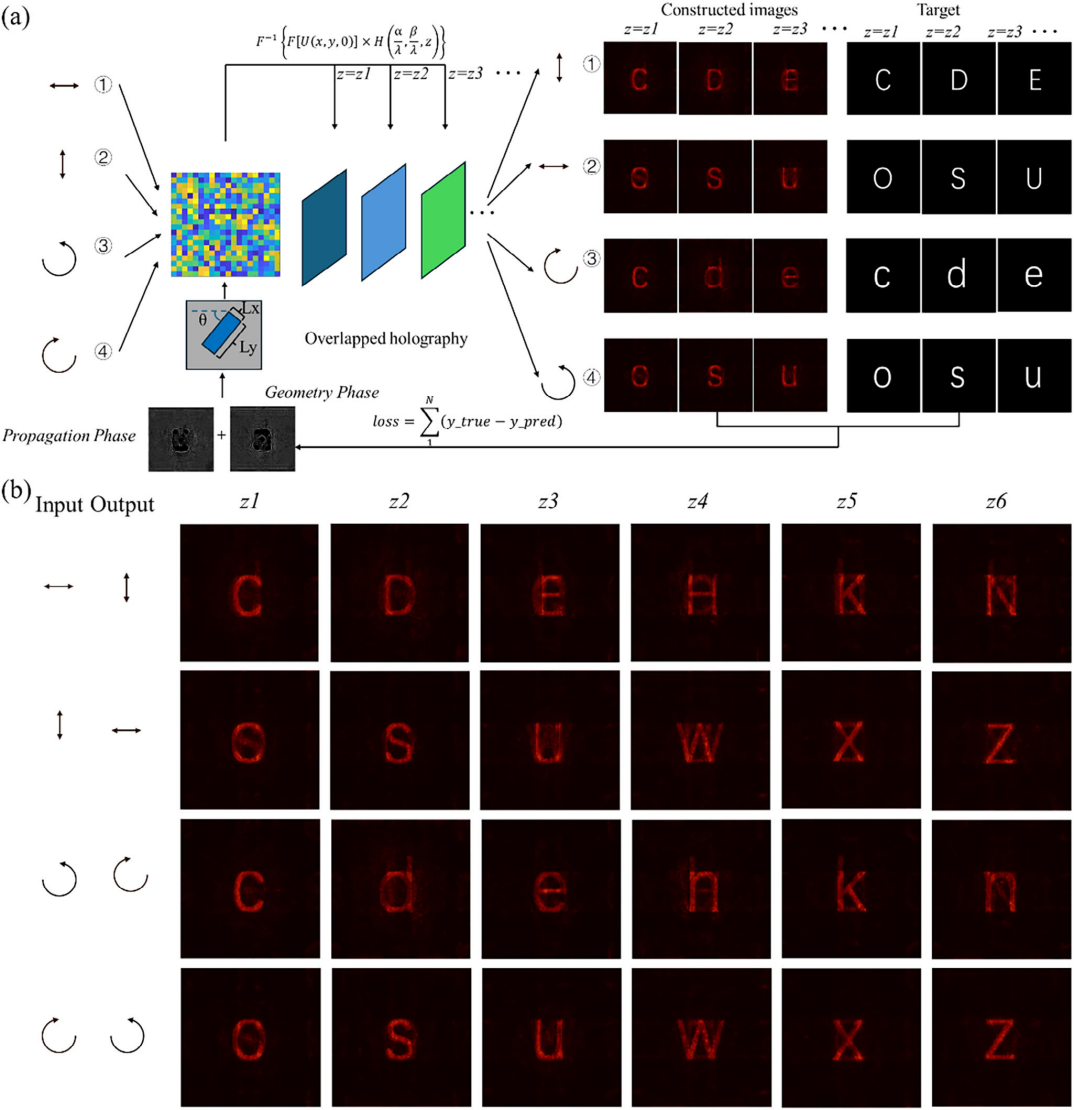
Simulated four polarization‐dependent 24 channels 3D display. a) The optimization flowchart for four polarization‐dependent 24 channels 3D display, the propagation distance is set at *z_1_* =3 mm, *z_2_* =5 mm, *z_3_* =7 mm, *z_4_* = 9 mm, *z_5_* =11 mm, *z_6_* =13 mm, the incident polarization is set as 0° LP, 90° LP, LCP, RCP, and analyzer polarization is set as 90° LP, 0° LP, RCP, LCP. b) The simulated construction holographic images for four polarization‐dependent 24 channels 3D display, the propagation distance is set at *z_1_* =3 mm, *z_2_* =5 mm, *z_3_* =7 mm, *z_4_* = 9 mm, *z_5_* =11 mm, *z_6_* =13 mm, the incident polarization is set as 0° LP, 90° LP, LCP, RCP, the filter polarization after the holographic construction is set as 90° LP, 0° LP, RCP, LCP.

We verified the polarization‐dependent 3D display both in simulation and experiment. Thanks to the large‐area and high‐efficiency fabrication method, we could design the polarization‐dependent 3D holographic metasurface with more pixel numbers. In the experiment above, eight different unit cells are used to demonstrate the fabrication ability of nanoprinting while reducing the design complexity because the LCP and RCP 3D display phases could be designed independently. However, as mentioned above, the incident polarization state we induce is just two, namely, LCP and RCP, which is insufficient for the increasing demand for multi‐dimensional 3D display and encryption 3D display, in other aspect, the nanoprinting fabricated metasurface provide an efficient way to increase the device area, and the inherent characteristic of the metasurface, including the size variation and rotation variation, undoubtedly greatly increase the design freedom by gradient descent method. Based on this, we introduce more polarizations in the 3D holographic display by gradient descent algorithm, as shown in **Figure** **6**a. In this optimization, the polarizer and analyzer are applied simultaneously to make holographic 3D images appear. When one particular polarization incident incidents, after the angular spectrum propagation, the mixed holographic images at different construction distances are filtered by analyzer and compared with the corresponding object images, in our four polarization‐dependent 3D holography simulation, we set the incident as 0° LP, 90° LP, LCP, RCP, and the corresponding analyzer polarization state are 90° LP, 0° LP, RCP, LCP, respectively, the matrix size of phase profile is set as 4500×4500. The difference of all four individual holographic 3D channels is calculated and the summation of all the differences is optimized in each iteration in the whole gradient descent process to decrease the loss. After 1500 iterations, all the 3D holographic images appear in different polarizations at different positions as shown in Figure 6b, proving that the global optimization is an efficient way to conceal more 3D information under different polarizations. By this powerful gradient descent method and high‐throughput nanoprinting metasurface, the polarization state could be further added by adding the matrix size of the phase profile. Our efforts provide an efficient way both in design and fabrication for multi‐polarization holographic 3D display, enabling smooth 3D holographic displays under different polarizations in the future.

## 3 Conclusion

In conclusion, to meet the increasing demand for enhanced information storage and large‐scale commercialization in 3D holographic imaging, we applied a modern design methodology, leveraging the ADAM gradient descent algorithm. This approach integrates polarization as an additional degree of freedom, thereby introducing a new dimension to electromagnetic wave manipulation. Through simulation and experiment, we successfully generated ten polarization‐dependent 3D holographic images using just 4000 × 4000 pixels by a high‐throughput metasurface. To make full use of the propagation phase and geometry phase modulation ability to generate more polarizations in 3D holographic display, we also take the simulation with twenty‐four 3D holographic images under four polarization states, the construction results prove that with the propagation phase and geometry phase applied in the design strategy, the 3D holographic display could be diverse with different polarizations. By combining advanced nanoprinting techniques with the ADAM optimization algorithm, we can substantially increase pixel density in both simulations and experiments, achieving smoother 3D objects at various polarizations. With the TiO_2_ particle‐doped resin in nanoprinting, all these designs could come into practice with a large scale. Our work successfully merges innovative design strategies with cutting‐edge nanoprinting and new materials, paving the way for the commercialization of high‐dimensional, practical 3D holographic displays in real‐world applications.

## 4 Experimental Section

### Gradient Descent Optimization

The ADAM algorithm was employed to optimize the phase patterns for polarization‐dependent 3D holography. In this work, phase values of the hologram are optimized, the phase profiles are initialized randomly in the range [0, 2π] across the hologram and are iteratively updated using gradient descent with Adam optimizer at a learning rate of 0.01. The loss function is defined as Equation (3).

For efficiently computing gradients with respect to numerous input variables, automatic differentiation is leveraged—a method that applies the chain rule to compute exact partial derivatives through a sequence of known operations. Within this framework, the forward problem, i.e., scalar diffraction, is first simulated. From this simulation, a computational graph is automatically constructed, comprising elementary operations for which gradients are analytically defined. As a result, a single forward pass suffices to compute gradients with respect to all optimization variables, offering significant advantages over traditional finite‐difference methods that require multiple evaluations per variable.

Once the gradient (∇_ϕ_ loss) is obtained, the optimization variables—modulation phase ϕ(x, y)—are iteratively updated in the direction of steepest descent. In this work, the forward simulation, automatic differentiation, and gradient‐based updates are implemented in Python v3.9 using the TensorFlow v2.10.0 framework. Each optimization run is terminated when the relative change in the loss function falls below 10^−5^ for 100 consecutive iterations, or after 1500 iterations, whichever comes first.

### Numerical Simulation

The numerical simulation is conducted by Ansys Lumerical *FDTD Solutions*, the periodic boundary condition is applied for both x‐ and y‐ directions, and for z‐ direction, the perfectly matched layer is used. To ensure consistency with the practical TiO_2_ particle‐doped resin, the measured refractive index (as depicted in Figure 3b) was used as the material parameter for the nanopillars in the unit cell. The substrate of the unit cell was set as quartz, with a refractive index of 1.45.

### Sample Fabrication

The polarization‐dependent 3D holographic display samples are fabricated by high‐throughput nanoprinting. To be specific, the whole nanoprinting process includes three procedures: First, the fabrication of the Si master mold. Second, the pattern transforms to the PET substrated soft mold. Third, the pattern transforms to the TiO_2_ particle‐doped resin‐based samples, this transformation process could be executed repeatedly. In the first Si master mold fabrication process, photoresist AR‐6200 is spin‐coated on a 2‐inch Si wafer, the opposite pattern is formed by electron beam exposure, after development, 50 nm thickness Cr is deposited on the photoresist via electron beam evaporation system (ULVAC ei‐5z), then liftoff is applied by acetone for the sample and the Cr hard mask is formed. The sample is then etched using inductively‐coupled plasma reactive ion etching (LEUVEN INSTRUMENTS), residual Cr is removed by Cr etchant and the Si master mold is finally completed. In the first pattern transform process from the Si master mold to the PET substrated soft mold, liquid PS is coated on the Si master mold, then the PET substrate is placed onto the coated PS layer, and the PS is solidated under UV illumination at the contact pressure 5000 Pa and then the PS is combined with the PET substrate. In the second pattern transform process from PS‐based soft mold to the quartz substrate, the final high‐throughput UV‐NIL transform process is automatically completed via a commercial NIL equipment (GL8/12 CLIV Gen2, GermanLitho GmbH).

## Conflict of Interest

The authors declare no conflict of interest.

## Supporting information



Supporting Information

## Data Availability

The data that support the findings of this study are available from the corresponding author upon reasonable request.
