# Supplementary material for: Polarization‐Dependent 3D Holography Generated by Inverse Design Nanoprinting Metasurface
Source: Adv Sci (Weinh). 2025 Dec 22;13(11):e19147. doi: 10.1002/advs.202519147 (PMC12931190; doi:10.1002/advs.202519147)
Supplement: Supplementary file 1 — Supporting Information [file ADVS-13-e19147-s001.docx]

Supporting Information

**Polarization-dependent 3D holography generated by inverse design nanoprinting metasurface**

*Lingxing Xiong, Jintao Gong, Fei Zhang*, Wenhao Miao, Qiong He, Dapeng Zhang, Yangjian Cai, Nan Chi**

L. Xiong, N. Chi

Key Laboratory for Information Science of Electromagnetic Waves (MoE), Fudan University, Shanghai 200433, China

Email: nanchi@fudan.edu.cn

L. Xiong, F. Zhang, W. Miao, D. Zhang, Q. He

National Key Laboratory of Optical Field Manipulation Science and Technology, Chinese Academy of Sciences, Chengdu 610209, China.

Email: zf@ioe.ac.cn

Fei Zhang, W. Miao, D. Zhang

College of Materials Sciences and Opto-Electronic Technology, University of Chinese Academy of Sciences, Beijing 100049, China

L. Xiong, F. Zhang, W. Miao

Research Center on Vector Optical Fields, Institute of Optics and Electronics, Chinese Academy of Sciences, Chengdu 610209 China

J. Gong, Y. Cai

Shandong Provincial Engineering and Technical Center of Light Manipulation, School of Physics and Electronics, Shandong Normal University, Jinan 250014, China

1. **The design principle of four polarization twenty-four channel 3D holography**

In the pursuit of multi-polarization 3D holography, the nanoprinting metasurface serves as a practical device to achieve this aim. First, we introduce the Jones matrix for the unit cells of the nanoprinting metasurface to clearly show our design strategy. We assume the Jones matrix of the unit cells of the metasurface as equation (1):

$$\text{J}=\left[\begin{matrix} A & B \\ C & D \end{matrix}\right]=\left[\widehat{e_{1}},\widehat{e_{2}}\right]^{-1}\left[\begin{matrix} \Lambda_{1} & 0 \\ 0 & \Lambda_{2} \end{matrix}\right]\left[\widehat{e_{1}},\widehat{e_{2}}\right]$$

To acquire different 3D holographic display in different polarizations, two polarizers and two quarter wavelength plates are put before and after the designed metasurface with propagation phase and geometry phase, thus, the arbitrary input ***p_i_*** and ***p_j_*** are added to the equation (1), thus the response of this metasurface under two polarizers and two quarter wavelength plates can be written as:

$$O_{ij}(x,y)=\langle p_{j}|J|p_{i}\rangle$$

Where (x,y) refers to the different positions on the metasurface. With the traditional design strategy, the single-layer metasurface is a reciprocal system, thus in equation (1), B=C, it simplifies the analysis complexity, but for these in-plane symmetric nanopillars, the linear characteristic of the eigen-polarization limits the input-output reactions to linear orthogonal polarization bases in conventional scenarios. To break through the bottleneck induced by the traditional design method, we take the performance of whole metaatoms into consideration in the multi-input and multi-output 3D holography, with the help of the ADAM gradient descent algorithm, all the sizes and rotation angles of the metaatoms are fine-tuned. To clarify the mechanism of our design, we combine the equation (1) and the equation (2), thus the output of the unitatom in different position could be rewritten as follows:

Where ^†^ represents the transpose and conjugate operation. Since the system we used is unitary,$\left[\widehat{e_{1}},\widehat{e_{2}}\right]^{-1}=\left[\widehat{e_{1}},\widehat{e_{2}}\right]^{\dagger}$, thus the equation (3) could be transform as follows:

Where $p_{i}^{'}$ and $p_{j}^{'}$ refer to the ***p_i_*** and ***p_j_*** interactive with the eigenvectors. To show our polarization-dependent strategy more clearly, we transform the linear orthogonal basis to the circular orthogonal basis, as shown in equation (5).

From this equation, the manipulation matrix could be decomposed into two parts, the eigen and conjugate components, namely, $J_{eigen}=\left[\begin{matrix} A_{LL} & 0 \\ 0 & A_{RR} \end{matrix}\right]$, $J_{con}=\left[\begin{matrix} 0 & B_{LR} \\ C_{RL} & 0 \end{matrix}\right]$. The A_LL_/A_RR_ and B_LR_/C_RL_ represent the co- and cross-polarization transformation under the circular based calculation method. For the expression of $|p_{i}\rangle=a_{1i}|L\rangle+a_{2i}|R\rangle$ and$\langle p_{j}|=a_{3j}^{*}\langle L|+a_{4j}^{*}\langle R|$, the various decomposition of input polarization state and the output polarization state could be achieved, proving that our design strategy to achieve multi-polarization 3D holographic display is feasible theoretically.

In our 24-channel four polarization state 3D holography simulation, the response of the metaatoms could be shown as follows:

$$\begin{matrix} \text{O}(x,y)=\left[p_{1},p_{2},p_{3},p_{4}\right]^{\dagger}\cdot J(x,y)\cdot\left[p_{1},p_{2},p_{3},p_{4}\right] \\ \text{=}\left[\begin{matrix} O_{1,1}(x,y) & O_{1,2}(x,y) & O_{1,3}(x,y) & O_{1,4}(x,y) \\ \sim& O_{2,2}(x,y) & O_{2,3}(x,y) & O_{2,4}(x,y) \\ \sim& \sim& O_{3,3}(x,y) & O{}_{3,4}(x,y) \\ \sim& \sim& \sim& O_{4,4}(x,y) \end{matrix}\right] \end{matrix}$$

With the different combinations of the input polarization state and the output polarization state, the unit cells at different positions on the metasurface show different responses. After the integral of the response with all the metaatoms, the global response is acquired. By this analysis, the global response of the metasurface could filter different holographic images under different input-output polarization states. In one specific input-output polarization state, the mixed electric field is transformed into the compomnent at this polarization state, and by the angular spectrum method, the holographic image at designed propagation distance is compared to the target image and the sum of all these differences decrease in every iteration in the gradient descent network. In the set of four batches of 24 object images at six different diffraction distances with a specific input-output polarization state in the gradient descent network, the 24 channel polarization-dependent 3D holography is finally acquired. According to equation (6), our design strategy makes full use of the metaatoms to achieve the 3D holography with the same input-output and the different input-output polarization states, significantly expanding the conventional dimension of the Jones matrix.

1. **The comparison for the maximum constructed holographic image number of 3D holography with traditional design method and ADAM gradient descent algorithm**


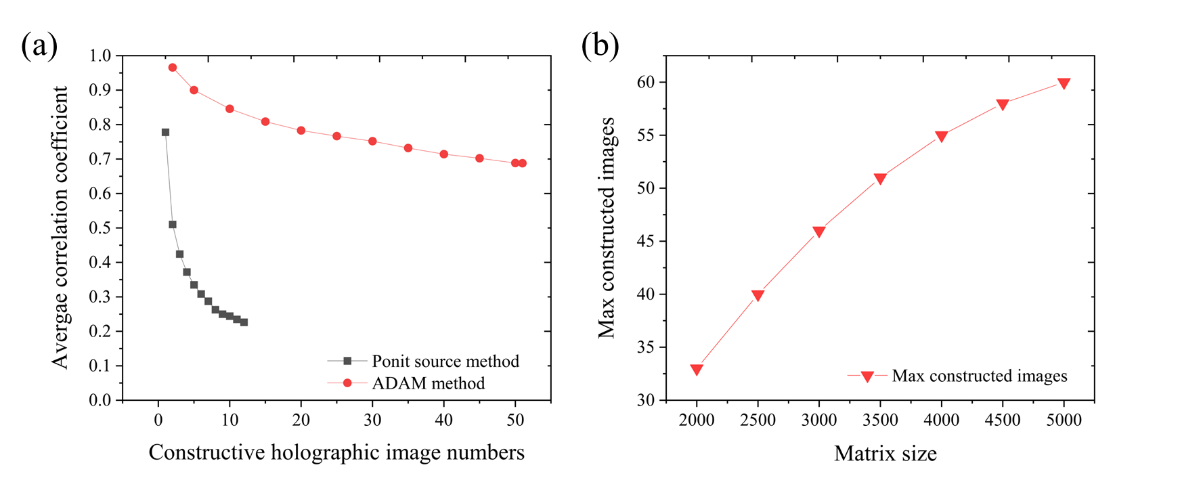


**Figure S1.** a) The average correlation coefficient for traditional point source method and ADAM gradient descent algorithm designed 3D holography. b) The maximum constructive holographic image number for 3D holography with the variation of simulated pixel number under the ADAM gradient descent algorithm, the correlation coefficient of all constructed holographic images is set above 0.6.

To further investigate the advantages of ADAM gradient descent algorithms applied in 3D holography, we conducted the simulations to calculate the average correlation coefficient of the whole constructed holographic images with the fixed matrix size (3500×3500) and gradually increased the simulated image number, the propagation distance started as 3 mm and the interval of adjacent construction is set as 500 μm for both two design methods. As shown in **Figure S1**a, the average correlation coefficient for ADAM gradient descent algorithm designed 3D holography is above 0.6 when the holographic image number is set as 51, comparing with the traditional point source method for the optimization of 3D holography, the average correlation coefficient decreases below 0.5 when the construction is set as 3, the high average correlation coefficient of the ADAM gradient descent algorithm designed 3D holography indicates that the ADAM gradient descent algorithm is a powerful tool to achieve multi-plane 3D holography.

To further investigate the maximum 3D holographic image number which constructed by the ADAM gradient descent algorithm, we fixed the correlation coefficient above 0.6 for every constructed image and gradually increased the pixel number from 2000×2000 to 5000×5000, the simulation results are shown in Figure S1b. After fitting the point between the number of maximum construction images and the pixel size, the following equation could be obtained.

$$N=-\frac{1}{500000}Pixel\_num^{2}+\frac{23}{1000}Pixel\_num-5,\left(Pixel\_num<5750\right)$$

where *N* is the maximum number of the 3D construction images under this ADAM gradient descent algorithm. The pixel number in this ADAM gradient descent algorithm for 3D holography is *Pixel_num*×*Pixel_num*. The ADAM gradient descent algorithm undoubtedly provides a powerful method for smooth 3D holography display with high calculation efficiency.

1. **The performance of unit cells based on TiO_2_ nanoparticles doped resin**


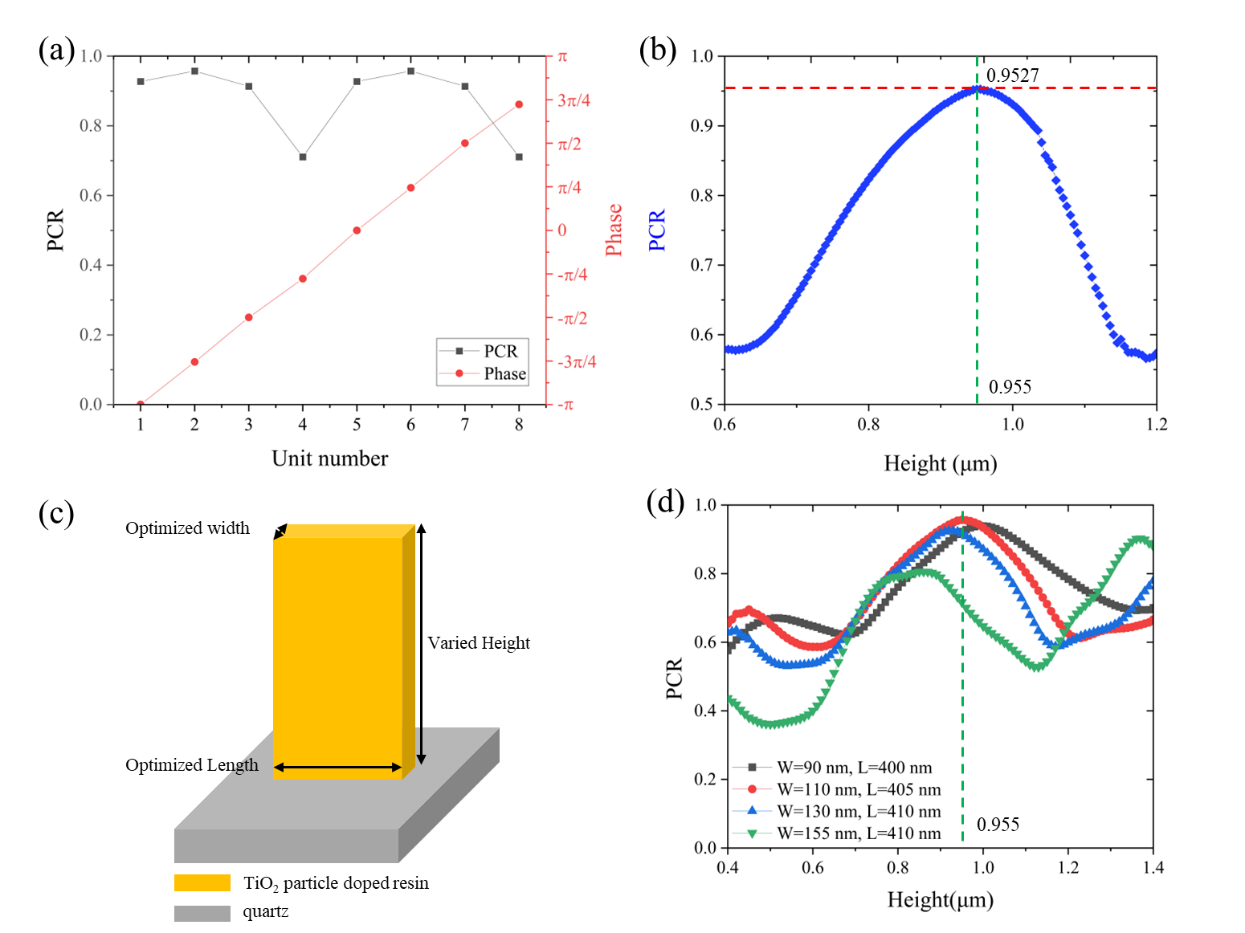


**Figure S2.** a) The propagation phase delays and PCRs of eight selected meta-atoms. b) Height optimization result of a typical meta-atom with L = 400 nm, W = 110 nm, and P = 450 nm. c) Schematic illustration of the eight optimized TiO_2_ particle-doped resin-based unit cells for height verification. d) The polarization conversion ratio of TiO_2_ particle-doped resin-based nanopillar with varied height, the four TiO_2_ particle doped resin based nanopillars with the size of *W* = 90 nm and *L* = 400 nm, *W* = 110 nm and *L* = 405 nm, *W* = 130 nm and *L* = 410 nm, *W* = 155 nm and *L* = 410 nm.

**Table S1.** Simulation results of PCR and propagation phase for 532 nm light incidence

| Unit | 1 | 2 | 3 | 4 | 5 | 6 | 7 | 8 |
| --- | --- | --- | --- | --- | --- | --- | --- | --- |
| Lx | 400 | 405 | 410 | 410 | 90 | 110 | 130 | 155 |
| Ly | 90 | 110 | 130 | 155 | 400 | 405 | 410 | 410 |
| Phase(°) | -179.91 | -135.95 | -89.91 | -49.92 | 0.00 | 44.05 | 90.09 | 130.08 |
| PCR | 0.9274 | 0.9574 | 0.9135 | 0.7107 | 0.9274 | 0.9574 | 0.9135 | 0.7107 |

To achieve polarization-dependent 3D holographic display through a high-throughput approach, nanoprinting emerges as a promising fabrication method for metasurfaces. Thus the performance of TiO_2_ particles doped resin with high refractive index is verified in both simulation and experiment. We first measured the refractive index of the TiO_2_ nanopracticle-doped resin and performed the unit cell sweep to find eight unit cells capable of covering 2π phase manipulation range according to the fabrication limitation. The working wavelength was set as 532 nm, thus the period is set as 450 nm accordingly. To minimize the fabrication complexity, the height of the nanopillars should be reduced but the lower of the nanopillar height could lead to the decrease of polarization conversion efficiency. Then we conducted the height optimization of TiO_2_ particles resin based nanopillars with width 400 nm and length 110 nm, the optimization result is shown in **Figure S2**b, when the height of TiO_2_ particles doped resin based nanopillar is set as 955 nm, the polarization conversion ratio reached 95.27%, considering the fabrication ability of electron beam lithography, the aspect ratio 10.6 is feasible and the height of the TiO_2_ particles doped resin based nanopillars is fixed at 955 nm. After the acquisition of the optimized height of TiO_2_ nanoparticles doped nanopillars, we should find eight unit cells which could not only cover the 2π transmission phase range but also maintain a relatively high polarization conversion ratio. Thus we conduct the sweep of length and width of the TiO_2_ particles doped nanopillars, the simulation result is shown in Figure 3c and 3d, and the TiO_2_ particles doped resin based eight unit cells which could meet the aforementioned conditions are picked out as shown in Figure S2a, the selected eight TiO_2_ nanoparticles doped resin based unit cells cover the 2π transmission phase range, the polarization conversion ratio of all eight unit cells is above 71.07%, to clearly show the transmission phase and the polarization conversion ratio, the values of all eight TiO_2_ nanoparticles doped resin based unit cells are shown in Table S1.

To further verify the performance of the eight optimized TiO_2_ particle-doped resin-based nanopillars with a height of 955 nm, we calculated the polarization conversion ratio of the TiO_2_ nanopillars with fixed width and length, while the height of the nanopillars is varied from 0.4 μm to 1.4 μm. For symmetry, the four unit cells with L=400 nm and W=90 nm, L=405 nm and W=110 nm, L=410 nm and W=130 nm, L=410 nm and W=155 nm are selected, as depicted in Figure S2c. After numerical calculations by FDTD Solution, the polarization conversion ratio is clearly shown in Figure S1d. From simulation results in Figure S2d, all four unit cells with L=400 nm and W=90 nm, L=405 nm and W=110 nm, L=410 nm and W=130 nm, L=410 nm and W=155 nm possess high polarization conversion ratio (>70%), it consistent with the polarization conversion ratio of the unit cell with L=400 nm and W=110 nm. For the symmetry, we could easily conclude that all eight optimized unit cells show a high polarization conversion ratio above 70%, with a height of 955 nm, the height greatly balances the fabrication complexity and the unit performance, the excellent performance of the optimized TiO_2_ nanoparticle-doped resin-based nanopillars indicates the nanoprinting metasurface could perform well in polarization-dependent 3D holography.

1. **Fabrication flowchart of high-throughput polarization-dependent 3D holography nanoprinting metasurface**


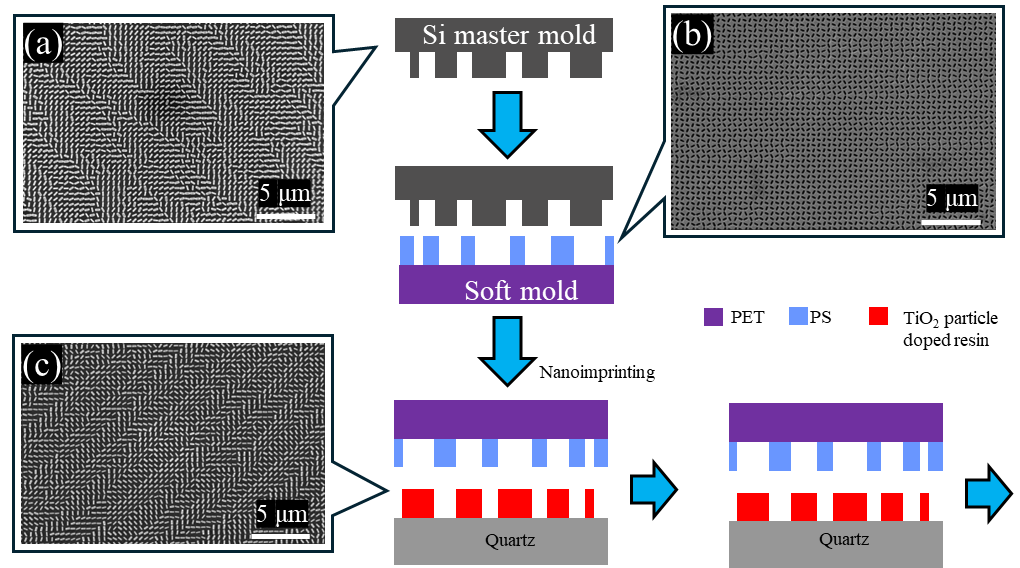


**Figure S3.** Schematic of the high-throughput fabrication process. a) SEM image of the first-generation rigid template (namely, Si master mold). b) SEM image of the second-generation flexible template (namely, PS soft mold). c) SEM image of the final polarization-dependent holographic meta-display replica.

With the consideration of production cost, the 2nd-generation soft mold and the final metasurface-based polarization-dependent 3D holography replicas are fabricated in batch mode using low-cost, high-throughput ultraviolet nanoprinting lithography (UV-NIL). The whole fabrication process consists of three processes, first, the creation of silicon (Si) master mold, namely the 1^st^ generation rigid template. Second, the replication of the soft polystamp (PS) mold, referred to as the 2^nd^ generation soft template. Third, the production of the final polarization-dependent 3D display items, as depicted in **Figure S3**c.

For the first Si rigid template fabrication procedure, electron beam writing (Elionix F125) is applied to generate a pattern that is an exact match to the target polarization-dependent 3D meta-display. Initially, a layer of photoresist (AR-6200) is spin-coated on the silicon wafer, with an inverse pattern being transferred to the photoresist through electron beam exposure. Then a 50 nm thick chromium (Cr) layer is deposited on the sample via the electron beam evaporation system (ULVAC ei-5z). Subsequently, a lift-off process is applied in acetone to define a Cr hard mask on the sample. After the lift-off process, the sample undergoes etching along the Cr mask using inductively coupled plasma reactive ion etching (LEUVEN INSTRUMENTS). The Si master mold is considered complete after removing any residue from the Cr hard mask by employing a Cr etchant (nitrate wet etching). In the next phase, liquid photosensitive PS resin is evenly spread over the Si master mold, and a PET substrate is placed on top of the coated PS film. The synthetic soft mold, composed of the PET substrate and the PS structures, is then solidified under ultraviolet light irradiation while maintaining a contact pressure of 5000 Pa. Subsequently, the soft mold and the Si template are carefully separated at a controlled, gradual pace. Finally, the high-throughput UV-NIL manufacturing process for polarization-dependent 3D meta-display replicas is automatically executed using a commercial NIL equipment (GL8/12 CLIV Gen2, GermanLitho GmbH). The scanning electron microscope (SEM) images amplified from the process flowchart depict the first-generation Si rigid template, the second-generation PS soft mold, and the desired polarization-dependent 3D meta-display replica (i.e., final product), respectively.

1. **The time consumption, cost, and production efficiency for different fabrication methods**

To show the advantages of the nanoprinting method for metasurface fabrication in throughput, cost for mass replication, and performance consistency across replicas, we compare the time consumption and the cost of the nanoprinting to the traditional electron beam lithography (EBL), focused ion beam (FIB), the detail is shown in Table S2.

**Table S2.** The time consumption and cost of per metasurface with different fabrication methods

|  | Nanoprinting | EBL | FIB |
| --- | --- | --- | --- |
| Cost per sample (millimeter level) | 2 USD (5.45 mm^2^) | Hundreds USD level | Hundreds USD level |
| Resolution | Based on the master mold (20 nm in this work) | 20 nm | 20 -50 nm |
| Throughput (mm^2^/min) | >3.63 | About 0.017 | About 6×10^-3^ |
| Consistency | same as master mold | cannot guarantee consistency | cannot guarantee consistency |
| Ref. | This work | [1] | [2] |

In our nanoprinting metasurface fabrication, the most cost is the TiO_2_ particle-doped resin, because the final nanoprinting metasurface could be completed by the softmold, in theory, the Si master mold could create infinite softmolds, thus, the expense of the fabrication of the Si master mold is not included. After assessing the total expense of the completion of the traditional method designed and the ADAM gradient algorithm designed polarization-dependent 3D holographic nanoprinting metasurface, only 2 USD for each replica. The morphology with all replicas is determined only by the initial Si master mold, it guarantees the consistency of all nanoprinted polarization-dependent 3D holographic metasurfaces. In our fabrication, the total time consumption of the ten replicas is less than 15 min, thus the pattern formation velocity is larger than 3.63 mm^2^/min. Compared with electron beam lithography (EBL) and focused ion beam (FIB) for the formation of comparable resolution patterns, the cost is hundreds level USD, and the pattern formation velocity is just about 0.017 mm^2^/min and about 6×10^-3^ mm^2^/min for EBL and FIB. To fabricate metasurfaces with traditional EBL and FIB, every metasurface is completed separately, thus the morphology of the batch-fabricated metasurfaces could be easily influenced by the equipment conditions and environmental parameters.

1. **Analysis of the influence of the residual layer on the performance of TiO_2_ particle-doped resin-based unit cells**


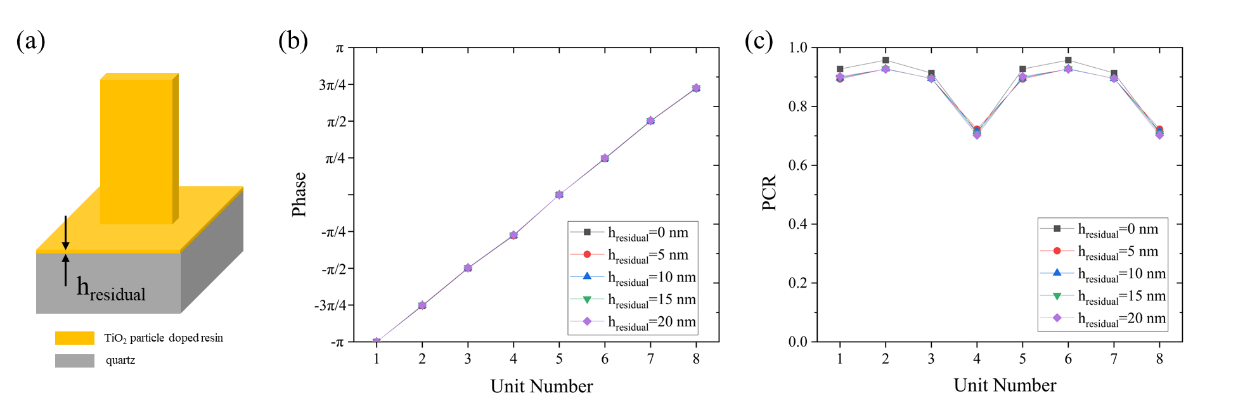


**Figure S4.** a) Schematic illustration of TiO_2_ particle-doped resin-based unit cells with a residual layer. b) The propagation phase of eight optimized TiO_2_ particle-doped resin-based unit cells with a residual layer, the height of the residual layer is set as 0 nm, 5 nm, 10 nm, 15 nm, 20 nm, respectively. c) The polarization conversion ratio of eight optimized TiO_2_ particle-doped resin-based unit cells with a residual layer, the height of the residual layer is set as 0 nm, 5 nm, 10 nm, 15 nm, 20 nm, respectively.

As a high-throughput fabrication method, nanoprinting metasurface is completed after two times pattern transformation, namely, from the Si master mold to the PET-based softmold, and from the PET-based softmold to the final TiO_2_ particle-doped resin-based metasurface. Thus, in the second pattern transformation from the PET-based softmold to the final sample, the TiO_2_ particle-doped resin is injected into the holes in the PET-based softmold, the TiO_2_ particle-doped particle based resin coated on quartz unavoidably remains a very thin layer on the quartz. Usually, the thin residual layer on the quartz is about 5 nm in height with measurement, but for consolidating the performance of the optimized unit cells with a large fabrication tolerance, we conduct the simulation to set the TiO_2_ particle-doped resin residual layer with heights of 0 nm, 5 nm, 15 nm, and 20 nm, respectively. As shown in **Figure S4**a, the residual is coated on the whole quartz substrate, and the width, length and height of the residual layer remain the same as the eight optimized unit cells. After simulation, the propagation phase of the eight optimized unit cells with residual layer is shown in Figure S4b, the propagation phase of the eight optimized unit cells with residual layer seems unchanged compared with the eight optimized unit cells without residual layer, and the eight units with 5 nm, 10 nm, 15 nm, 20 nm height residual layer cover 2π range, proving that the residual layer coated optimized unit cells show great phase modulation ability. For the polarization conversion ratio in Figure S4c, the polarization conversion ratio show a minor descent, about two percent for the unit cell with L=400 nm and 90 nm, 405 nm and 110 nm, 410 nm and 130 nm, L=90 nm and W=400nm, L=110 nm and W=405 nm, L=130 nm and W=410 nm, but the polarization conversion ratio is all above 89%, and the polarization conversion ratio for the optimized unit cell with L =410 nm and 155 nm, 155 nm and 410 nm remain almost unchanged. It further proves that the excellent polarization conversion ability for the optimized unit cells when the residual layer exists. The excellent polarization conversion ability and the phase modulation ability with different height residual layers illustrate that the nanoprinting is a robust fabrication method and could tolerate minor fabrication errors.

1. **Analysis of the influence of truncated rectangular pyramid morphology on the performance of the TiO_2_ particle-doped resin-based nanopillars**


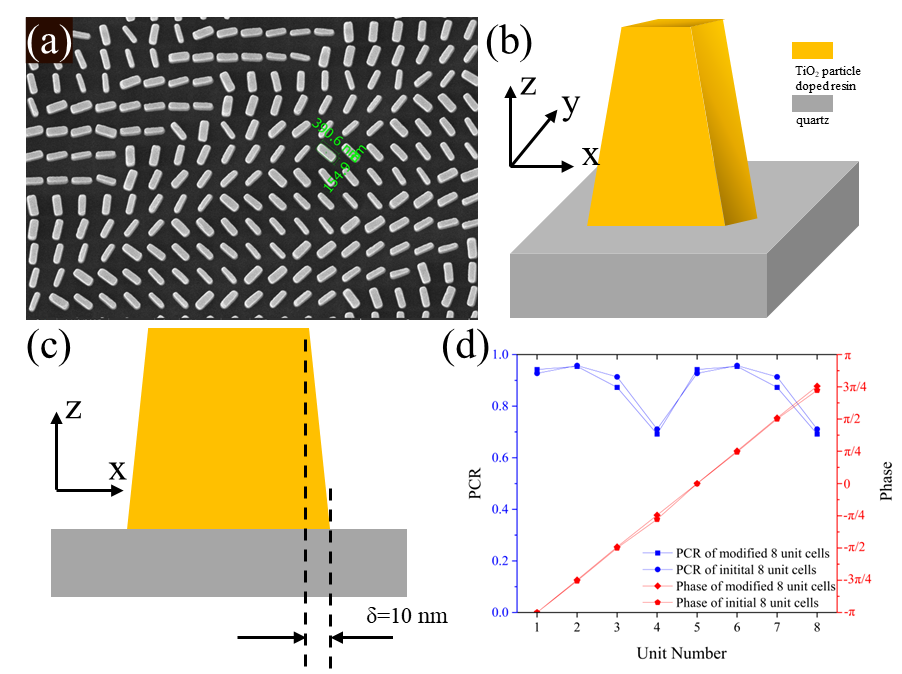


**Figure S5.** a) Measurement of the top size of one selected optimized unit cells (designed length = 410 nm, designed width = 155 nm) in scanning electron microscope (SEM) images. b) Perspective view of the truncated rectangular pyramid morphological TiO_2_ particle-doped resin-based nanopillar. c) Front view of the truncated rectangular pyramid morphological TiO_2_ particle-doped resin-based nanopillar. d) The Transmission phase and the polarization conversion ratio of the truncated rectangular pyramid morphological TiO_2_ particle doped resin based nanopillar.

In the first procedure of the nanoprinting, namely, the fabrication of Si master mold. After EBL pattern forming, Cr deposition, development, ICP etching, and Cr removing, the Si master mold was finally complete. With our designed height of TiO_2_ particle doped resin based nanopillars is set as 955 nm, by inductively coupled plasma reactive ion etching to form the same morphology as the final sample in Si, the etching gas is not uniform at different etching depths with a totally 955 nm etching depth, thus the top size of the nanopillars should be a little smaller compared to the design size. To investigate the top size deviation and the top size deviation influence on the performance of the designed optimized eight unit cells, we measured the top size of one optimized unit cell (designed length: 410 nm, designed width: 155 nm) and simulated the performance of other seven designed optimized TiO_2_ particle-doped resin-based nanopillars according to this deviation ratio. As shown in **Figure S5**a, the measured top size of the fourth optimized unit cell is 390.6 nm in length and 154.9 nm in width, compared to the designed size, the top width of the selected unit cell in the naoprinting metasurface remain almost unchanged, and the deviation ratio of the length of the top size of the selected unit cell is 4.73%, thus we remain the width of the eight optimized TiO_2_ doped resin based nanopillars unchanged, and fix the top length deviation ratio of 5% for eight optimized TiO_2_ doped resin based nanopillars to calculate the polarization conversion ratio and the propagation phase of the modified optimized unit cells. The perspective view and the front view of the modified eight optimized TiO_2_ particle-doped resin-based nanopillars are shown in Figure S5b and Figure S5c, respectively. After simulation, the polarization conversion ratio and propagation phase are shown in Figure S5d. From Figure S5d, the polarization conversion ratio of truncated rectangular pyramid morphological eight TiO_2_ particle doped resin based nanopillars with a negligible decrease compared with the corresponding unit cells as designed size, and the propagation phase of truncated rectangular pyramid morphological eight TiO_2_ particle doped resin based nanopillars almost unchanged compared to the corresponding unit cells as designed size, the propagation phase covers 2π range either. The simulation results prove that the minor fabrication deviation of the top size of the nanopillar has almost no influence on its performance, the excellent phase modulation ability and polarization conversion ability with minor fabrication deviation of top size deviation further solidifies the robustness of the nanoprinting method. The simulated raw data (PCR and propagation phase) of the truncated rectangular pyramid morphological TiO_2_ particle-doped resin-based nanopillars are shown in Table S3.

**Table S3.** Simulation results of PCR and propagation phase for the truncated rectangular pyramid morphological TiO_2_ particle-doped resin-based nanopillars with 532 nm light incidence

| Unit Number | 1 | 2 | 3 | 4 | 5 | 6 | 7 | 8 |
| --- | --- | --- | --- | --- | --- | --- | --- | --- |
| Designed length | 400 | 405 | 410 | 410 | 90 | 110 | 130 | 155 |
| Designed width | 90 | 110 | 130 | 155 | 400 | 405 | 410 | 410 |
| Modified length (nm) | 380 | 384.75 | 389.5 | 389.5 | 90 | 110 | 130 | 155 |
| Modified width (nm) | 90 | 110 | 130 | 155 | 380 | 384.75 | 389.5 | 389.5 |
| PCR | 0.9420 | 0.9543 | 0.8728 | 0.6919 | 0.9420 | 0.9543 | 0.8728 | 0.6919 |
| Phase | -180 | -134.56 | -88.44 | -44.11 | 0 | 45.44 | 91.55 | 135.89 |

1. **Additional simulation and experiment results for polarization-dependent 3D holography**

To perfectly show the 3D imaging ability of the TiO_2_ particles doped polarization-dependent 3D holography nanoprinting metasurface, we compare the holographic images at different propagation distances between simulation and experiment. In simulation, we set the holographic images, number “3”, the letter “D”, and the IOE logo with a focus at the diffraction distances 3 mm, 4 mm, 5 mm, respectively. The simulated constructive images by angular spectrum method are clearly distinguished at the propagation distances 3 mm, 4 mm, 5 mm, respectively, as shown in **Figure S6**a. To show the holographic images change with the propagation distance, an experiment is conducted to show the different holographic images at different propagation distances, as shown in Figure S6b. Three designed images are clearly displayed at the propagation distances 3 mm, 4 mm, 5 mm, respectively, proving the perfect 3D holographic display ability of the nanoprinted metasurface.


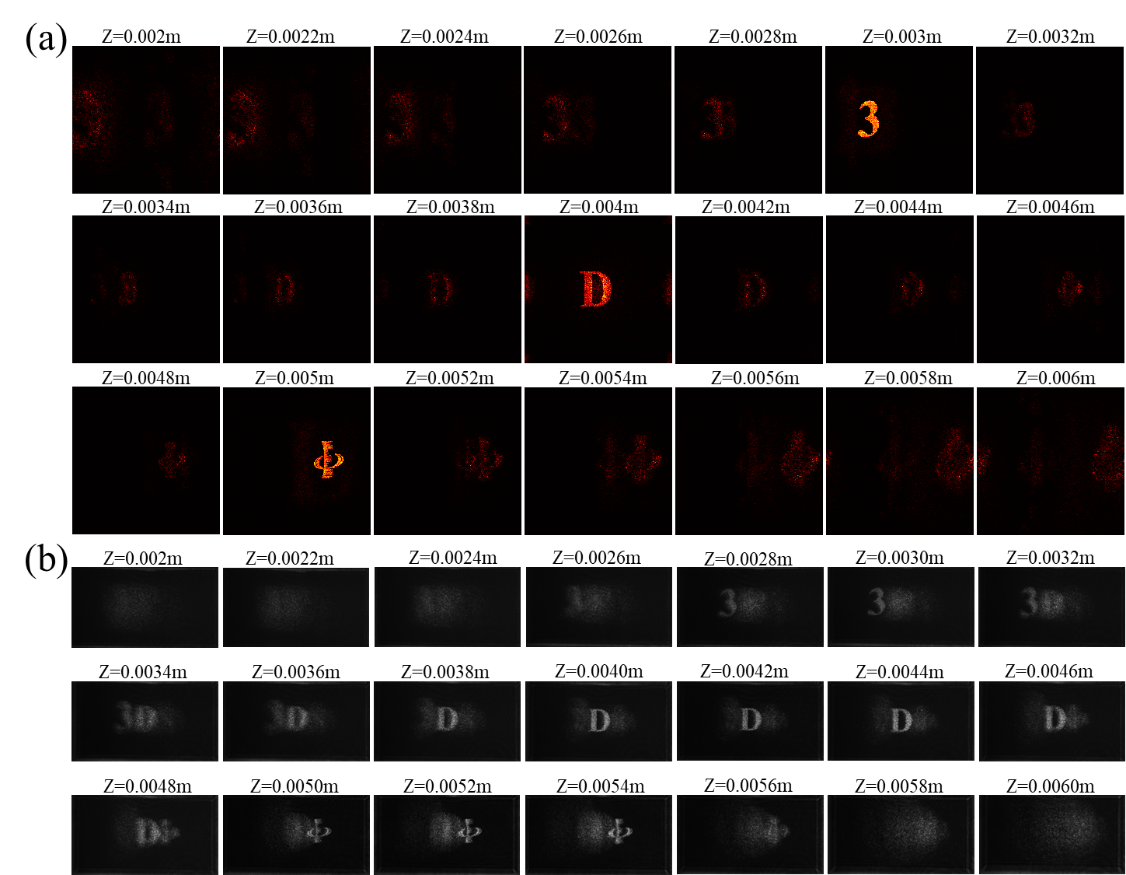


**Figure S6.** a) Simulation results for the construction of holographic images at the propagation distance from 2 mm to 6 mm, the interval distance between adjacent constructive holographic images is set as 0.2 mm. b) Experimentally captured holographic images at the distance from 2 mm to 6 mm, the interval distance between adjacent measured holographic images is set as 0.2 mm.

1. **Optical setup for polarization-dependent 3D holography based on nanoprinting metasurface**

**
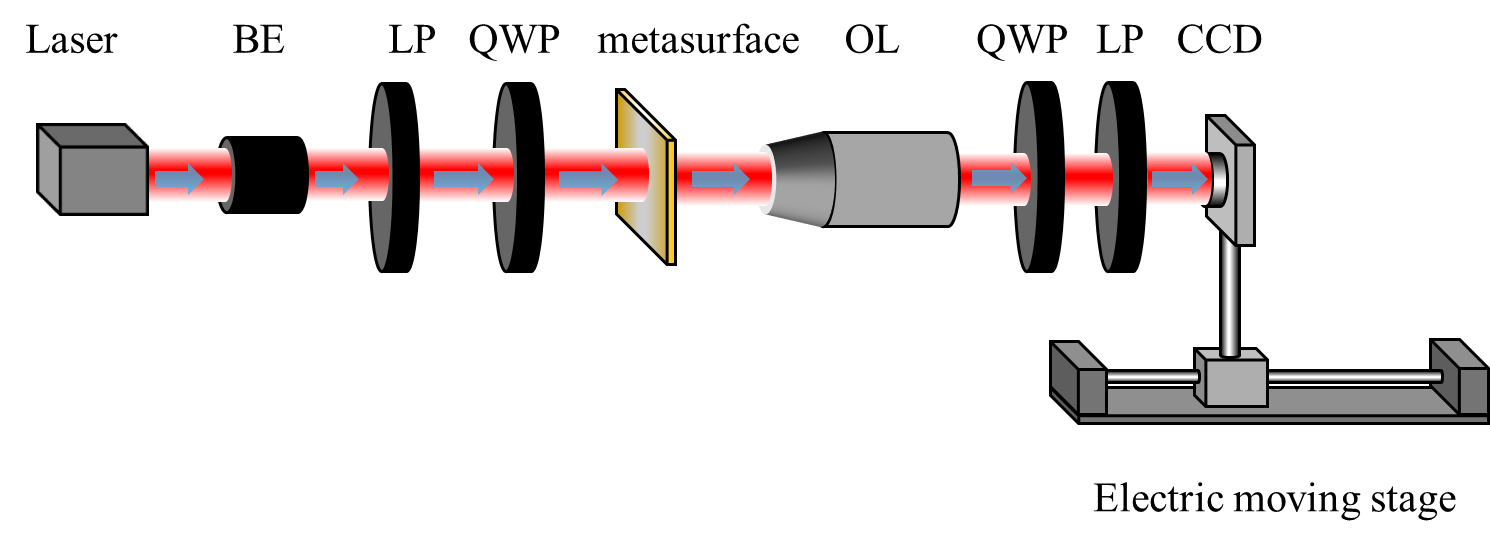
**

**Figure S7.** Schematic illustration of the optical measurement setup for polarization-dependent 3D holography based on nanoprinting metasurface (BE: beam expander, LP: linear polarizer, QWP: quarter waveplate, OL: object lens, CCD: Charge Coupled Device).

As shown in **Figure S7**, a continuous laser is utilized as source in the experiment. The laser beam successively passed through a beam expander, a linear polarizer, a quarter waveplate, the polarization-dependent holographic 3D meta display metasurface, an object lens, a quarter waveplate, a linear polarizer and finally reached the CCD. The object lens placed in front of the CCD is used to make the holographic enlarged and captured by CCD (2048 × 2048 pixels, GT2050NIR, Allied Vision), which features a pixel size of 5.5 × 5.5 µm. Notably, the visible to near-infrared CCD camera was assembled on an electronically controlled movement platform with a displacement accuracy of 1 µm, and it can be moved automatically along the stage track by using the controller software on a host computer.

1. **Efficiency of Polarization-dependent 3D holography based on high-throughput nanoprinting metasurface**

To fully characterize the holography quality for nanoprinting metasurface-generated polarization-dependent 3D holography, we measured the power of every holographic images under different polarizations and distances, the measuring optical setups are shown in Figure S7, the only difference is the CCD replaced by a power meter. The beam from the laser go through the beam expander, first LP and QWP, thus a LCP or RCP beam is incident on the nanoprinting metasurface, the second QWP and LP serve as the filter to abandon the same polarized light, then the power of the converted polarization beam was captured by the power meter. When we change the state of the second LP and QWP, the output polarization state could be altered to the same state as the beam incident on the nanoprinting metasurface. Thus, the efficiency was finally acquired. With the changing of the power meter position at the holographic imaging distance, all the efficiency of the polarization-dependent 3D holography was obtained. The efficiency of the traditional method designed and the ADAM gradient descent method designed holographic images based on the nanoprinting method is shown in Table S4 and Table S5, respectively.

**Table S4.** The efficiency of the traditional method designed nanoprinting metasurface generated polarization-dependent 3D holography

|  | LCP | | | RCP |
| --- | --- | --- | --- | --- |
| pattern | 3 | D | IOE logo | ship |
| Replica1 | 61.9% | 63.39% | 63.65% | 77.77% |
| Replica2 | 63.34% | 62.99% | 63.31% | 77.56% |
| Replica3 | 63.70% | 63.90% | 63.61% | 77.74% |

**Table S5.** The efficiency of the ADAM gradient descent method designed nanoprinting metasurface generated polarization-dependent 3D holography

| LCP | 3 | D | I | O | E |
| --- | --- | --- | --- | --- | --- |
| efficiency | 80.78% | 80.84% | 80.64% | 81.04% | 81.04% |
| RCP | F | U | D | A | N |
| efficiency | 80.61% | 80.92% | 81.01% | 81.32% | 81.58% |

Reference

[1] A. E. Grigorescu, C. W. Hagen, *Resists for sub-20-nm electron beam lithography with a focus on HSQ: state of the art,* *Nanotechnology* **2009**, *20*, 292001.

[2] Y. Kim, I. Kuljanishvili, *Progress in fabrication techniques and applications of patterned low-dimensional materials and nano-films,* *Nano Express* **2025**, *6*.
